# A strategy to design novel structure photochromic sensitizers for dye-sensitized solar cells

**DOI:** 10.1038/srep08592

**Published:** 2015-02-26

**Authors:** Wenjun Wu, Jiaxing Wang, Zhiwei Zheng, Yue Hu, Jiayu Jin, Qiong Zhang, Jianli Hua

**Affiliations:** 1 Key Laboratory for Advanced Materials and Institute of Fine Chemicals, East China University of Science & Technology, 130 Meilong Road, Shanghai, 200237, China

## Abstract

Two sensitizers with novel structure were designed and synthetized by introducing photochromic bisthienylethene (BTE) group into the conjugated system. Thanks to the photochromic effect the sensitizers have under ultraviolet and visible light, the conjugated bridge can be restructured and the resulting two photoisomers showed different behaviors in photovoltaic devices. This opens up a new research way for the dye-sensitized solar cells (DSSCs).

Among the various processes to utilize solar energy, DSSCs that are based on highly porous nanocrystalline films of titanium dioxide (TiO_2_) have received considerable attention due to their high power conversion efficiency, low cost, and high semiconductor stability^1^,^2^,^3^,^4^,^5^,^6^,^7^,^8^. To further improve their energy conversion efficiencies, much effort has been devoted to the optimization of components (e.g. sensitizers, electrolyte and counter electrodes) and to the design of creative novel structures of DSSCs^9^,^10^,^11^,^12^,^13^,^14^,^15^,^16^,^17^,^18^,^19^,^20^,^21^,^22^,^23^,^24^.

Pure organic dyes, as a major candidate of the sensitizers for DSSCs^25^,^26^,^27^,^28^,^29^,^30^,^31^,^32^,^33^,^34^,^35^,^36^,^37^,^38^,^39^,^40^,^41^,^42^,^43^,^44^,^45^,^46^,^47^,^48^,^49^,^50^,^51^,^52^, have been extensively explored with the basic D-π-A structure. The energy level of these dyes exerts a significant influence on the photovoltaic performances^53^. The conventional methods to adjust the orbital levels were to change their donors, π-bridges or acceptors^46^,^47^,^48^,^49^,^50^,^51^,^52^,^53^,^54^,^55^,^56^. It is well-known that photochromic compounds based on bisthienylethene (BTE) unit are one of the most promising materials because of their excellent fatigue resistance and thermal stability in both isomeric forms. The open- and closed-ring isomers of BTE differ from each other not only in their absorption but also in optical data storage and optical signal processing. In this work, we herein incorporated the photochromic BTE unit into D-π-A sensitizers in order to develop optical switching sensitizers for dye-sensitized solar cells. As shown in Figrue 1, the photochromic dyes based on BTE moiety (BTE-CA and BTE-CN) can form two photoisomers (coded as CNO and CNC or CAO and CAC with different acceptor) with the open or closed-ring by alternating irradiation with UV and visible light^57^,^58^,^59^,^60^. Therefore, the photoelectric conversion efficiency (PCE) of the DSSCs based on these dyes can be changed with the structure of the sensitizers tuned reversibly under irradiation of UV or visible light.

In Figure S1, CAO/CAC and CNO/CNC represent the open-ring/closed-ring forms of compounds BTE-CA and BET-CN, respectively. As shown in Figure 2a, upon alternating irradiation with UV and visible light, the sensitizers showed typical photochromic properties. When irradiated at 365 nm, the compounds showed a reduction in intensity of the absorption around 380 nm and a rise of a new absorption at 574 nm and 694 nm for BTE-CA and BTE-CN, respectively (Figure 2a and Figure S2). The low energy band appeared at 574 nm for CAC or 694 nm for CNC, arising from the charge-transfer transition, suggests the formation of large D-π-A conjugated closed-ring diarylethene, which corresponds to the colour change of the solution from colourless to bluish-purple or yellow to green (inset in Figure 2a). Comparing to CAC, the absorption spectrum of CNC extended into the near infrared region due to the stronger electron-withdrawing character of cyanoacetic acid group. Upon irradiation with visible light (λ > 500 nm), the bluish-purple or green solution bleached to colourless or yellow, indicating that the retrieving of open-ring isomer (CAO or CNO). After anchoring on TiO_2_ film, the λ_max_ of CAC and CNC hypsochromically shifted to 560 nm and 589 nm, respectively, which can be ascribed to the deprotonation and aggregation of the dyes (Figure 2b).

To obtain and characterise the molecular orbital energy levels, cyclic voltammetry (CV) was employed to measure the oxidation potential of the dyes in CH_2_Cl_2_; these CV curves are shown in Figure S3. The corresponding electrochemistry data are given in Table S1, and the energy levels are demonstrated in Figure S4. The first two oxidation potentials (*E*_ox_) of different isomers, corresponding to the highest occupied molecular orbital (HOMO) and HOMO-1 levels, were converted to a normal hydrogen electrode (NHE) with ferrocene/ferrocenium (Fc/Fc^+^) as an external reference. The zeroth-zeroth energy (*E*_0–0_) values, defined as the optical gap of the sensitizers, were obtained from the absorption thresholds (Table S1). From above data, we found that their HOMO and LUMO levels thermodynamically matched well with the iodine/iodide redox potential value (0.4 V) and *E*_cb_ of the TiO_2_ electrode (0.5 V vs. NHE).

Figure 3 shows the *J*-*V* and *P*-*V* curves with the corresponding photovoltaic data summarized in Table S1. From Figure 3 and Table S1, the photocurrent density *vs*. voltage curves for DSSCs based on CAO, CAC, CNO and CNC were given and these cells have a solar energy to electricity conversion efficiency of 0.87% (*J*_sc_ = 2.00 mA cm^−2^, *V*_oc_ = 602 mV, *ff* = 0.72), 0.30% (*J*_sc_ = 0.91 mA cm^−2^, *V*_oc_ = 500 mV, *ff* = 0.65), 2.00% (*J*_sc_ = 4.42 mA cm^−2^, *V*_oc_ = 650 mV, *ff* = 0.70), 0.59% (*J*_sc_ = 1.61 mA cm^−2^, *V*_oc_ = 540 mV, *ff* = 0.68), respectively. In these data, the short-circuit photocurrent (*J*_sc_) and *V*_oc_ are critical parameters determining the energy conversion efficiency of the cells. While *J*_sc_ is mostly controlled by the light-harvesting and charge-injection efficiency of sensitizer, *V*_oc_ is determined by the difference between the quasi-Fermi level in the TiO_2_ and the energy level of the redox couple in the electrolyte^61^. As we all know, the charge recombination between injected electrons and oxidized species in the electrolyte will result in a reduced *V*_oc_^62^,^63^,^64^,^65^,^66^,^67^,^68^,^69^,^70^,^71^.

To analyse why the sensitizers with an open-ring give better photovoltanic performances in DSSCs, the orbital distributions of different isomers (Figure S4) were achieved by density functional theory (DFT) calculations at the B3LYP/6-31G* level. As illustrated in Figure S4, the HOMO orbitals in CAO and CNO are primarily located at the π-framework of the donor part, while the electron density of the LUMOs are delocalized over the BTE unit and anchoring group. The distinct location of the HOMO and LUMO orbitals enables a good charge separation. However, for CAC and CNC, the electron density of HOMO or LUMO orbital locates at the conjunction bridges (BTE unit) and acceptors, suggesting the strong electron-donating ability of the closed-ring leads to a poor charge separation.

In summary, two new D-π-A type sensitizers for DSSCs based on BTE photochromic unit, BTE-CA and BTE-CN, were successfully synthesized and their photovoltaic performances were characterised. There are some elements for shaping their PCE performance including the variation of absorption spectroscopy, orbital distribution, and CB shift following the photochromic interconversion between the different photoisomers by alternating irradiation with UV and visible light. Using their tautomeric characteristics, we first attempt to achieve a regulation of the photovoltaic performance of the sensitizer with photons of different wavelengths.

## Author Contributions

**Figure 1:**
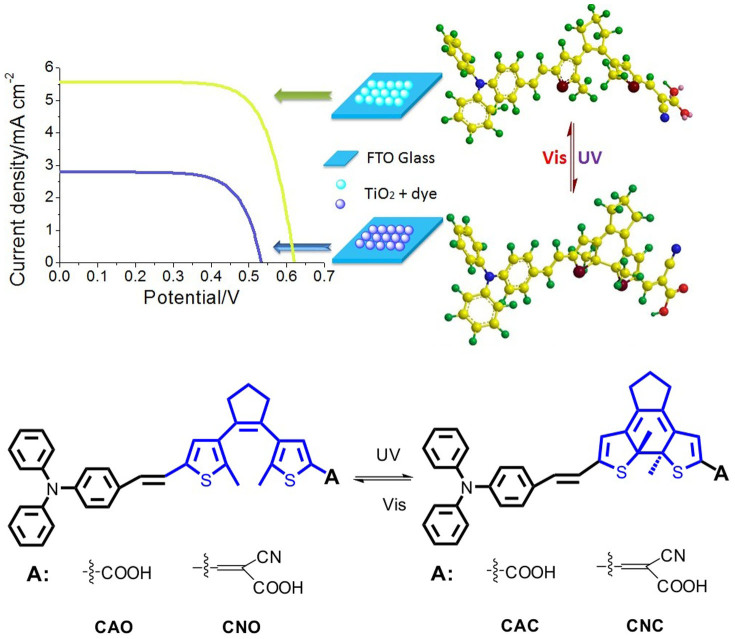
The restructure of photochromic dyes under UV or visible light and its influence on J-V curve.

**Figure 2:**
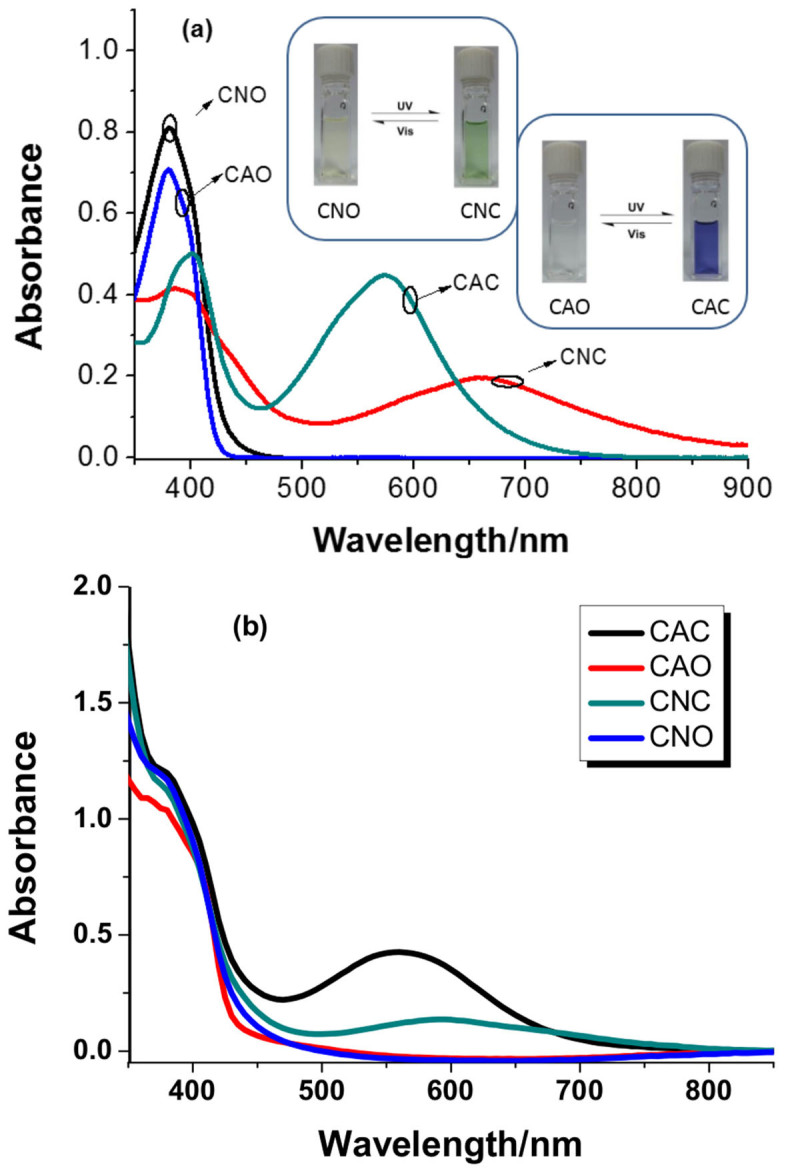
(a) UV-Vis absorption spectra of different photoisomers. Inset: Photographic images of interconversion between CAO and CAC or CNO and CNC under the alternative irradiation with UV or visible light in CH^2^Cl^2^ solution. (b) The absorption spectra of CAO, CAC, CNO and CNC on TiO^2^ film.

**Figure 3:**
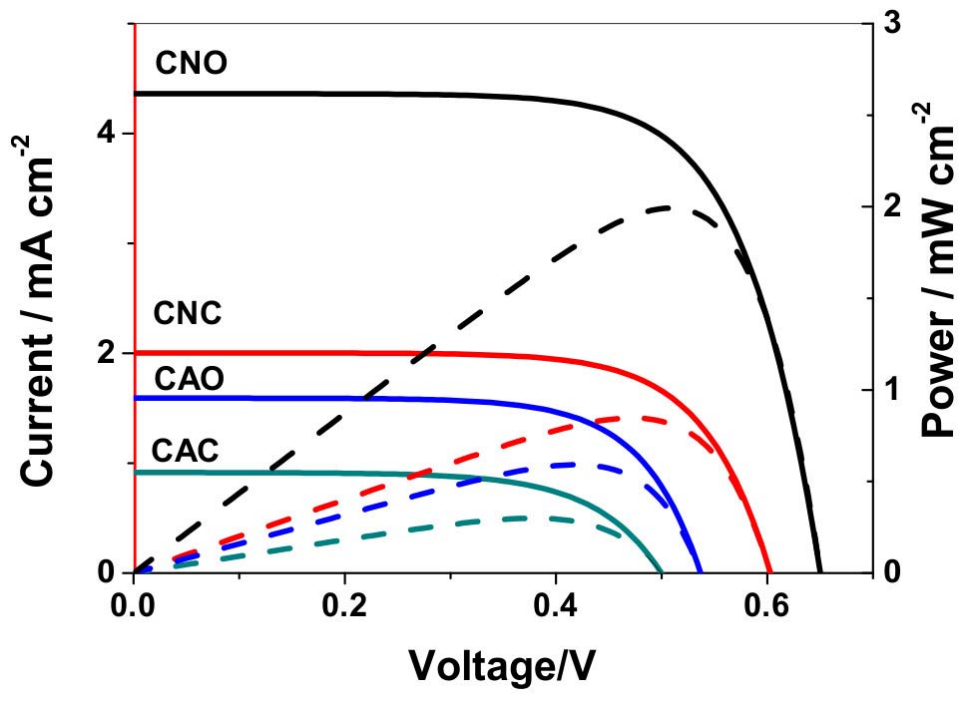
Photocurrent density and power vs. voltage curves of DSSCs based on different isomer of CAO/CAC or CNO/CNC under irradiation of AM 1.5 G simulated solar light (100 mW cm^−2^). Solid line: Photocurrent density vs. voltage; Dash line: Power vs. voltage.

W.J.W. wrote the main manuscript and Figure 1–3. J.X.W. and J.Y.J. synthesized the compounds. Z.W.Z. prepared the photovoltaic devices and characterized the photoelectric properties. Y.H. and Q.Z. provided the theoretical calculation and guidance. J.L.H. wrote parts of discussion section. All authors reviewed the manuscript.

## Supplementary Material

Supplementary Information Supplementary information
