# Supplementary material for: A strategy to design novel structure photochromic sensitizers for dye-sensitized solar cells
Source: Sci Rep. 2015 Feb 26;5:8592. doi: 10.1038/srep08592 (PMC4341208; doi:10.1038/srep08592)
Supplement: Supplementary Information [file srep08592-s1.pdf]

Supporting Information

A strategy to design novel structure photochromic sensitizers for dye-sensitized solar cells

Wenjun Wu, Jiaxing Wang, Zhiwei Zheng, Yue Hu, Jiayu Jin, Qiong Zhang, Jianli Hua*

Key Laboratory for Advanced Materials and Institute of Fine Chemicals, East China University of Science & Technology, 130 Meilong Road, Shanghai 200237, PR China

*Corresponding Author: wjwu@ecust.edu.cn

1. Experimental Section

1.1 Synthesis

All reactions were performed under the air atmosphere except the one with n-butyl lithium (1.6 M solution in hexane) which was purchasd from Sigma-Aldrich and used without further purification. Other starting materials were commercially available and purified before use. All other reagents were of analytical purity and used without further treatment. The synthetic pathways of the desired CAO and CNO are shown in Figrure S1.

Synthesis of 1-(5-chloro-2-methyl-3-thienyl)-2-(5-(triphenylamine-1-yl)vinyl-2-methyl-3-thienyl) cyclopen- tene (4)

A stirring solution of **3** (1.4 g, 4.5 mmol) and phosphonium salt (3.0 g, 5 mmol) in DMF (25 mL) was added C_2H_5ONa (0.34 g, 5 mmol). The mixture was then refluxed overnight. After cooling down to room temperature, water (50 mL) was added. The resulting mixture was extracted by dichloromethane (3 times) and the solvent was removed in vacuum. The residue was purified by column chromatography (silica gel,

petroleum ether) to give the compound **4** (0.92 g, 36.2%) as a yellow solid; ^{1}H NMR (400 MHz, $CDCl_3$) δ 7.31 – 7.23 (m, 5H), 7.09 (d, *J* = 7.6 Hz, 4H), 7.00-7.04 (m, 5H), 6.97 (d, *J* = 16.0 Hz, 1H), 6.69 (d, *J* = 16.0 Hz, 1H), 6.68 (s, 1H), 6.61 (s, 1H), 2.79 – 2.69 (m, 4H), 2.07 – 1.99 (m, 2H), 1.96 (s, 3H), 1.87 (s, 3H); ^{13}C NMR (101 MHz, $CDCl_3$) δ 147.56, 147.11, 139.11, 135.90, 135.15, 135.12, 133.80, 133.70, 133.28, 131.34, 129.30, 127.01, 126.88, 126.82, 126.77, 125.00, 124.47, 123.64, 123.01, 120.43, 38.42 (s, 5H), 22.91 (s, 3H), 14.62 (s, 2H), 14.23 (s, 3H); HRMS (ESI) calcd for $C_{35}H_{31}ClNS_2$ (M+H) 564.1586, found 564.1585

Synthesis of 1-(5-formyl-2-methyl-3-thienyl)-2-(5-(triphenylamine-1-yl) vinyl- 2-methyl-3-thienyl) cyclopean- tene (5)

Compound **4** (0.90 g, 1.60 mmol) was dissolved in anhydrous THF (15 mL) and n-butyl lithium (1.5 mL of 1.6 M solution in hexane) was added dropwise under nitrogen at 0 ℃ using a syringe. The mixture was then stirred at 0 ℃ for 30 mins and quenched with anhydrous dimethylformamide (0.62 mL). The mixture was stirred for one hour at room temperature, before pouring into H_2O. The mixture was extracted with ether. The organic layer was dried over $MgSO_4$, and concentrated. The residue was purified by column chromatography (silica gel, CH_2Cl_2/ petroleum ether 1:1) to give the compound **5** (0.29g, 32.6%) as a yellow solid; ^{1}H NMR (400 MHz, $CDCl_3$) δ 9.74 (s, 1H), 7.46 (s, 1H), 7.29-7.23 (m, 5H), 7.10 (d, *J* = 7.7 Hz, 5H), 7.02 (t, *J* = 8.0 Hz, 4H), 6.95 (d, *J* = 15.9 Hz, 1H), 6.69 (d, *J* = 16.0 Hz, 1H), 6.67 (s, 1H), 2.81 (t, *J* = 7.5 Hz, 4H), 2.12 – 2.04 (m, 2H), 2.08 (s, 3H), 1.92 (s, 3H); ^{13}C NMR (101 MHz, $CDCl_3$) δ 182.62, 147.52, 147.20, 146.65, 139.80, 139.54, 138.11, 137.77, 136.37, 135.54, 133.74, 133.05,

131.14, 129.30, 127.08, 127.03, 126.53, 124.49, 123.56, 123.05, 120.17, 38.46, 38.33, 23.02, 15.51, 14.55; HRMS (ESI) calcd for $C_{36}H_{32}NOS_2$ (M+H) 558.1925, found 558.1923

Synthesis of 1-(5-carboxy-2-methyl-3-thienyl)-2-(5-(triphenylamine-1-yl)vinyl-2-methyl-3-thienyl) cyclo- peantene (CAO)

Compound **4** (0.40g, 0.71 mmol) was dissolved in anhydrous THF (10 mL) and n-butyl lithium (0.67 mL of 1.6 M solution in hexane) was added dropwise under nitrogen at 0 ℃ using a syringe. After 30 mins, solid CO_2 (excess) was added. After 1 h, water (20 mL) was added. The mixture was acidified with 10% HCl, and then extracted with ether. The organic layer was dried over $MgSO_4$ and concentrated. The residue was purified by column chromatography (silica gel, ethyl acetate/ petroleum ether 1:3) to give a yellow solid (0.34 g, 83.5%). ^{1}H NMR (400 MHz, DMSO) δ 12.92 (s, 1H), 7.44 (s, 1H), 7.42 (d, J = 10.4 Hz, 1H), 7.32 (t, J = 7.9 Hz, 4H), 7.15 (d, J = 16.1 Hz, 1H), 7.05 (dd, J = 19.4, 7.5 Hz, 6H), 6.91 (d, J = 8.6 Hz, 2H), 6.87 (s, 1H), 6.70 (d, J = 16.1 Hz, 1H), 2.75 (t, J = 10.6 Hz, 4H), 2.05 – 1.99 (m, 2H), 1.97 (s, 3H), 1.86 (s, 3H); ^{13}C NMR (101 MHz, DMSO) δ 162.67 , 146.87, 146.50, 141.64, 138.77, 136.62, 135.69, 134.80, 133.94, 133.32, 132.90, 130.82, 130.23, 129.51, 127.24, 127.00, 126.43, 124.10, 123.89, 123.21 123.07, 122.82, 120.27, 37.97, 37.83, 22.26, 14.41, 14.08; HRMS (ESI) calcd for $C_{36}H_{32}NO_2S_2$ (M+H) 574.1874, found 574.1874

Synthesis of 1-(5-acrylicacidethenyl-2-methyl-3-thienyl)-2-(5-(triphenylamine-1-yl) vinyl- 2-methyl-3-thienyl) cyclopeantene (CNO)

A mixture of **5** (110 mg, 0.2 mmol), cyanoacetic acid (85 mg, 1.0 mmol), and ammonium acetate (77 mg, 1 mmol) in acetic acid was placed in a three-necked flask under a nitrogen atmosphere and was stirred at 120 ℃ for 12 h. After cooling, the reaction was quenched by adding water and then extracted with CH_2Cl_2. The organic layer was dried over anhydrous $MgSO_4$ and evaporated under vacuum. The products were purified by silica gel column chromatograph eluted with CH_2Cl_2/CH_3OH (6/1). The purple solid was isolated in 80% yield (98 mg, 0.16 mmol). ^{1}H NMR (400 MHz, DMSO) δ 7.87 (s, 1H), 7.77 (s, 1H), 7.43 (d, J = 8.0 Hz, 2H), 7.39 (s, 1H), 7.32 (t, J = 8.0 Hz, 4H), 7.16 (d, J = 16.0 Hz, 2H), 7.07 – 6.99 (m, 6H), 6.91 (d, J = 8.0 Hz, 2H), 6.87 (s, 1H), 6.70 (d, J = 16.0 Hz, 1H), 2.77 (t, J = 6.6 Hz, 4H), 2.04 – 1.99 (m, 2H), 1.98 (s, 3H), 1.87 (s, 3H). ^{13}C NMR (101 MHz, DMSO) δ 164.56, 146.97, 146.60, 141.72, 141.38, 138.89, 136.57, 135.78, 135.09, 133.14, 133.10, 133.06, 130.92, 129.64, 127.36, 127.13, 126.54, 124.22, 123.35, 122.92, 120.44, 118.84, 38.11, 22.57, 22.32, 14.57, 14.22; HRMS (ESI) calcd for $C_{39}H_{31}N_2O_2S_2$ (M-H) 623.1827, found 623.1828

DFT Calculations

The ground-state geometries of CNO, CNC, CAO and CAC have been optimized in the gas phase by DFT with the Gaussian09 package, [S1] using the hybrid B3LYP [S2] functional and the standard 6-31G(d) basis set. For the TDDFT calculations, performed on the B3LYP optimized ground-state geometries, the mPW1K (modified Perdew-Wang 1-parameter for kinetics) DFT exchange correlation functional of Truhlar and coworkers [S3] was used with 6-31G(d) basis set. Solvation effect was taken into account

into the TDDFT calculations in CH_2Cl_2 with the nonequilibrium version of the C-PCM model [S4] implemented in Gaussian09.[S1]

Characterisation

^{1}H NMR and ^{13}C NMR spectra in $CDCl_3$ were recorded on Brucker AM-400 spectrometers with tetramethylsilane (TMS) as the internal standard. Mass spectra (MS) were recorded on EI mass spectroscopy. UV-vis absorption spectra were performed on a Varian Cray 500 spectrophotometer. The current-density voltage (J-V) characteristics of the DSSCs were measured by recording J-V curves using a Keithley 2400 source meter under the illumination of AM1.5 G simulated solar light (Newport-91160 equipped with a 300 W Xe lamp and an AM 1.5 G filter). The incident light intensity was calibrated to 100 mW cm^{-2} with a standard silicon solar cell (Newport 91150V).

References

S1 Gaussian 09, Revision A.02, Frisch, M. J.; Trucks, G. W.; Schlegel, H. B.; Scuseria, G. E.; Robb, M. A.; Cheeseman, J. R.; Scalmani, G.; Barone, V.; Mennucci, B.; Petersson, G. A.; Nakatsuji, H.; Caricato, M.; Li, X.; Hratchian, H. P.; Izmaylov, A. F.; Bloino, J.; Zheng, G.; Sonnenberg, J. L.; Hada, M.; Ehara, M.; Toyota, K.; Fukuda, R.; Hasegawa, J.; Ishida, M.; Nakajima, T.; Honda, Y.; Kitao, O.; Nakai, H.; Vreven, T.; Montgomery, J. A.; Jr., Peralta, J. E.; Ogliaro, F.; Bearpark, M.; Heyd, J. J.; Brothers, E.; Kudin, K. N.; Staroverov, V. N.; Kobayashi, R.; Normand, J.; Raghavachari, K.; Rendell, A.; Burant, J. C.; Iyengar, S. S.; Tomasi, J.; Cossi, M.; Rega, N.; Millam, J. M.; Klene, M.; Knox, J. E.; Cross, J. B.; Bakken, V.; Adamo, C.; Jaramillo, J.; Gomperts, R.; Stratmann, R. E.; Yazyev,

O.; Austin, A. J.; Cammi, R.; Pomelli, C.; Ochterski, J. W.; Martin, R. L.; Morokuma, K.; Zakrzewski, V. G.; Voth, G. A.; Salvador, P.; Dannenberg, J. J.; Dapprich, S.; Daniels, A. D.; Farkas, O.; Foresman, J. B.; Ortiz, J. V.; Cioslowski, J.; Fox, D. J.; Gaussian, Inc., Wallingford CT, 2009.

S2 Becke, A. D. *J. Chem. Phys.* **1993**, 98, 1372–1377.

S3 Lynch, B. J.; Fast, P. L.; Harris, M.; Truhlar, D. G. *J. Phys. Chem. A* **2000**, 104, 4811–4815.

S4 Cossi, M.; Barone, V. J. *Chem. Phys.* **2001**, 115, 4708–4717.

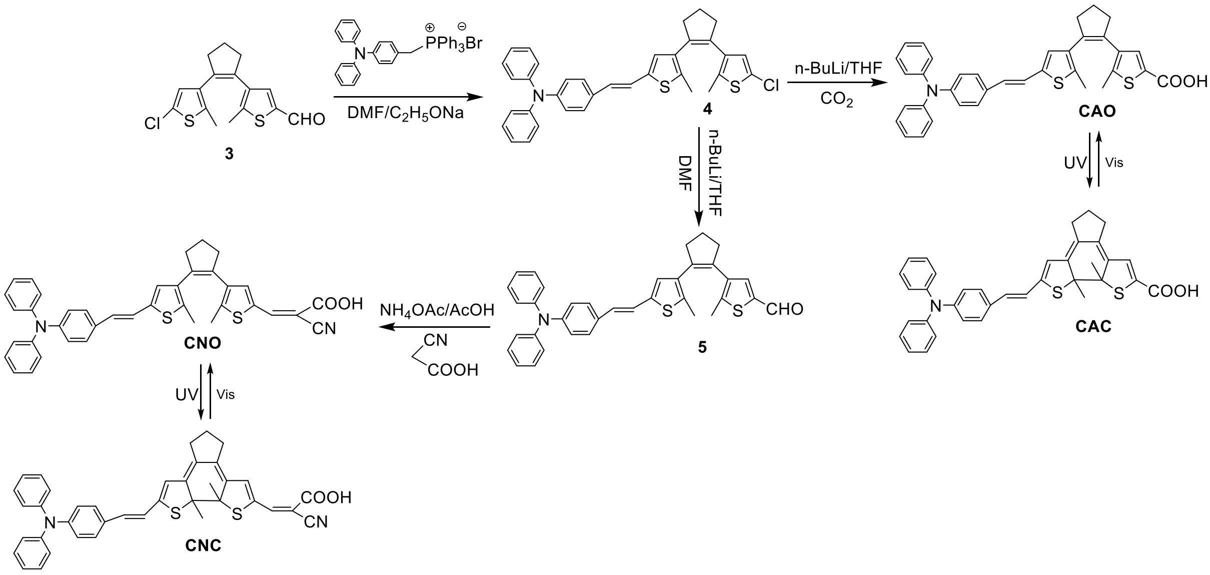

Figure S1. Synthetic route and interconversion of the photoisomers of CAO/CAC and CNO/CNC.

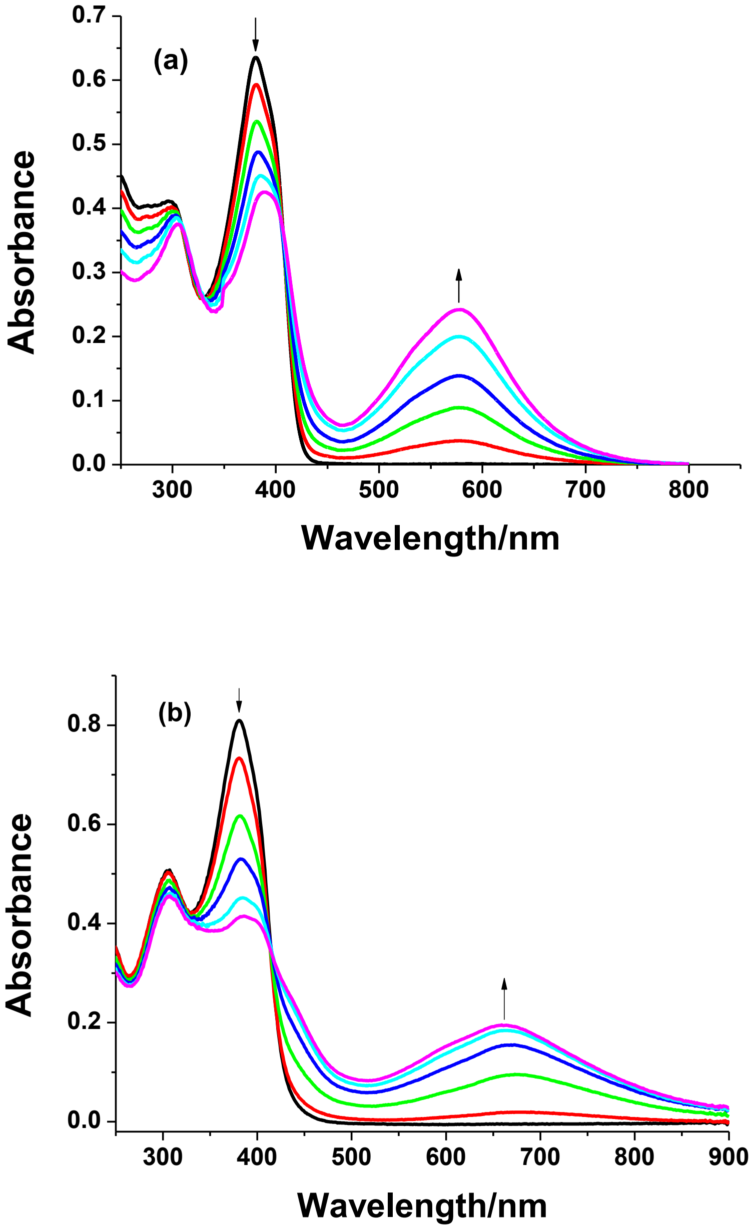

Figure S2. UV-vis spectral changes of the (a) CAO (2.0×10^{-5} M) (0-1 min) and (b) CNO (2.0×10^{-5} M) (0-40 min) upon 365 nm light irradiation in CH_2Cl_2.

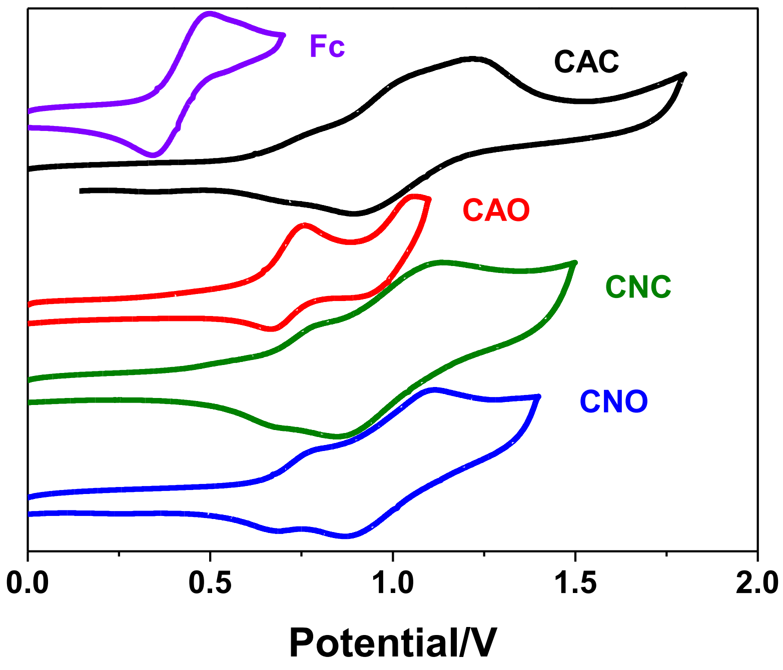

Figure S3. Cyclic voltammogram plots for CAO, CAC, CNO and CNC in dichloromethane solution with 0.1 M tetrabutylammonium hexafluorophosphate ($TBAPF_6$) as supporting electrolyte.

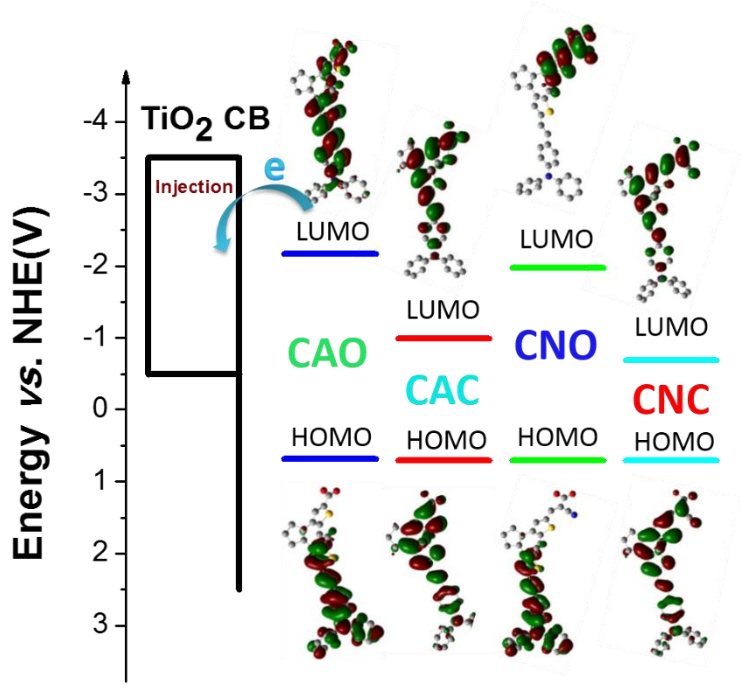

Figure S4. The calculated frontier molecular orbitals of HOMO and LUMO diagram for CAO, CAC, CNO and CNC.

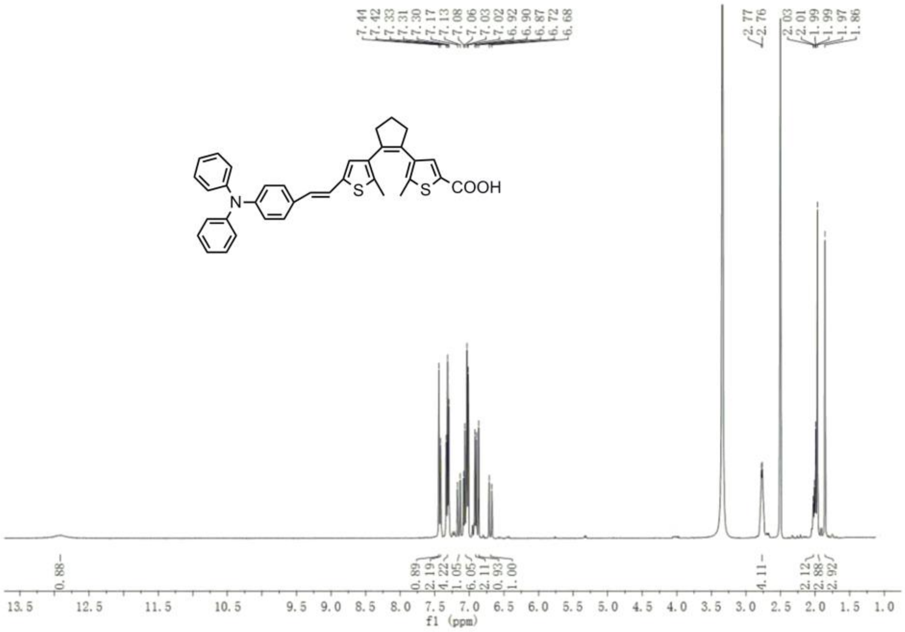

Figure S5. ^{1}H NMR (DMSO, 400 MHz) spectrum of compound CAO.

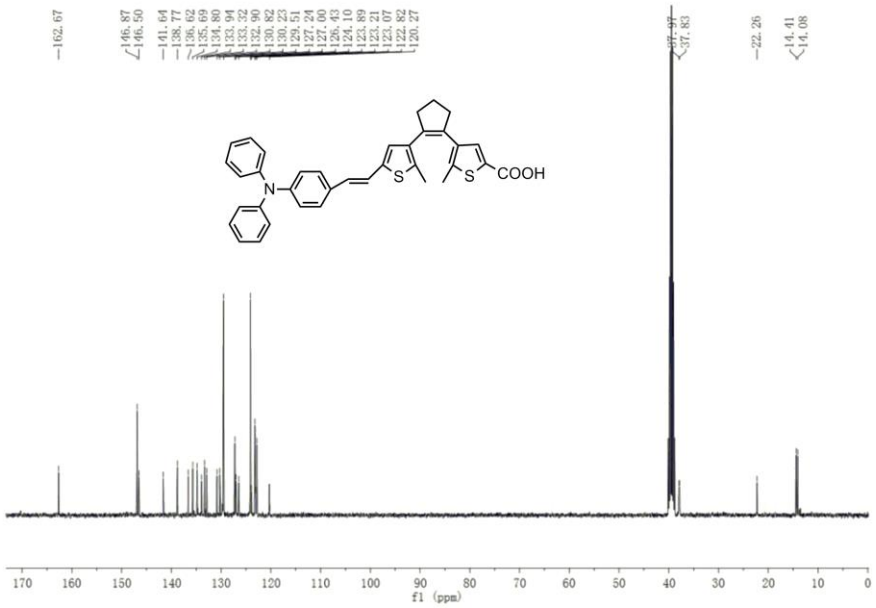

Figure S6. ^{13}C NMR (DMSO, 100 MHz) spectrum of compound CAO.

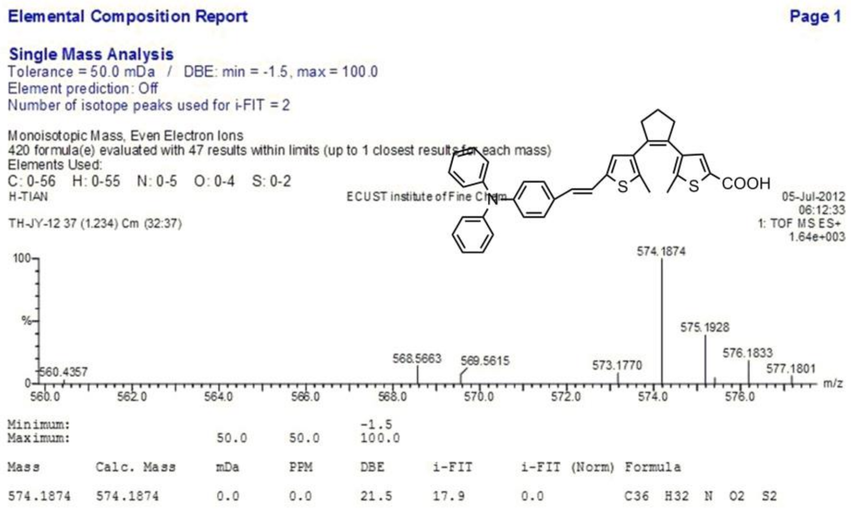

Figure S7. High resolution mass spectrum of CAO.

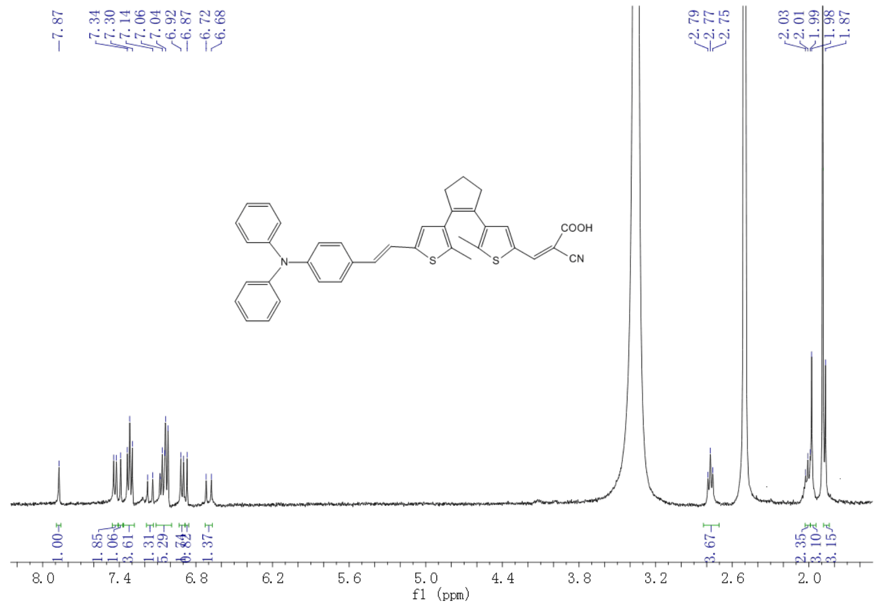

Figure S8. ^{1}H NMR (DMSO, 400 MHz) spectrum of compound CNO.

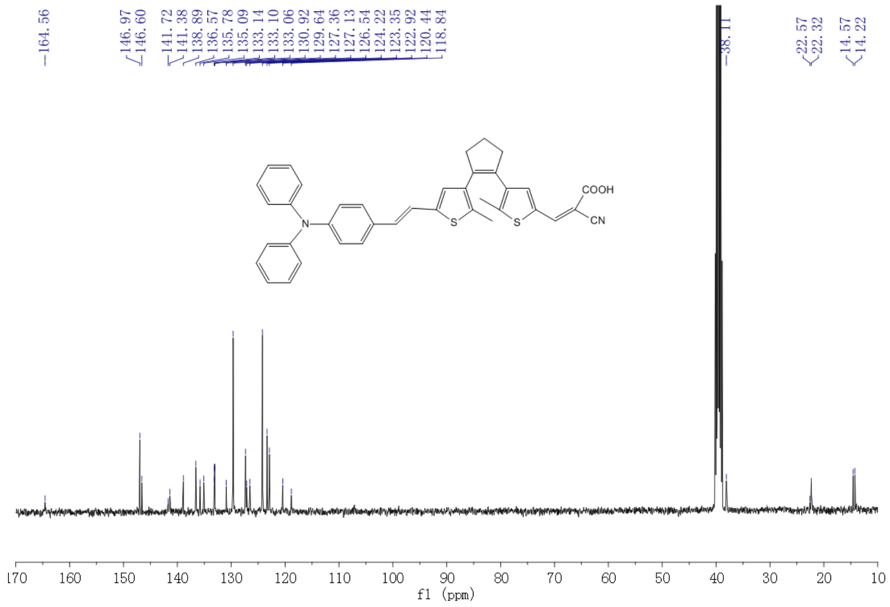

Figure S9. ^{13}C NMR (DMSO, 100 MHz) spectrum of compound CNO.

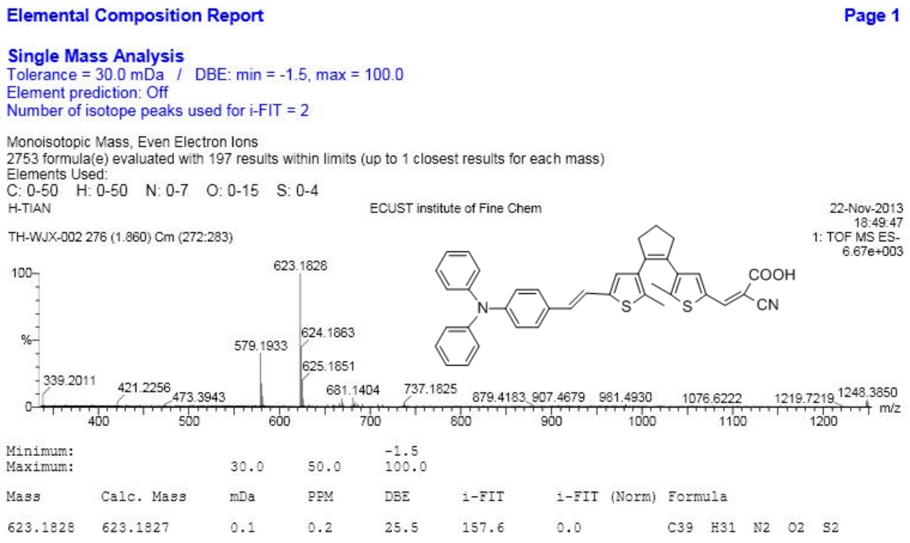

Figure S10. High resolution mass spectrum of CNO.

Table S1. Absorption, electrochemical and photovoltaic performance of DSSCs based on different isomers of BTE-CA or BTE-CN under irradiation of AM 1.5 G simulated solar light (100 mW cm^{-2}).

Isomer	$\lambda_{max}{}^{a}$/nm ($\varepsilon\times10^{-4}$ M^{-1} cm^{-1})		Potential and energy levels ^{b}			Photovoltaic performance			
			HOMO/V (vs. NHE)	E_{0-0}/eV	LUMO/V (*vs.* NHE)	V_{oc}/mV	J_{sc}/mA cm^{-2}	ff	PCE
CAO	380(3.54)		0.68	2.86	-2.17	602	2.00	0.72	0.87
CAC	401(2.50)	574(2.24)	0.71	1.71	-0.99	500	0.91	0.65	0.30
CNO	380(4.05)		0.71	2.68	-1.97	650	4.42	0.70	2.00
CNC	380(2.08)	659(0.98)	0.71	1.41	-0.70	540	1.61	0.68	0.59

[a] Absorption peaks (λ_{max}) and molar extinction coefficients (ε) of different photoisomers of CAO , CAC, CNO and CNC were measured in CH_2Cl_2 (2×10^{-5} M); [b] E_{0-0} values were estimated from the absorption thresholds from absorption spectra of dyes adsorbed on the TiO_2 films and LUMO is estimated by subtracting E_{0-0} from HOMO.
